# MLPA identification of dystrophin mutations and *in silico* evaluation of the predicted protein in dystrophinopathy cases from India

**DOI:** 10.1186/s12881-017-0431-6

**Published:** 2017-06-13

**Authors:** Sekar Deepha, Seena Vengalil, Veeramani Preethish-Kumar, Kiran Polavarapu, Atchayaram Nalini, Narayanappa Gayathri, Meera Purushottam

**Affiliations:** 1 Department of Neuropathology, Neuromuscular Laboratory, Bengaluru, Karnataka India; 2 Department of Neurology, National Institute of Mental Health and Neurosciences, Bengaluru, Karnataka India; 3 Molecular Genetics Laboratory, Neurobiology Research Centre, Department of Psychiatry, National Institute of Mental Health and Neurosciences, Hosur Road, Bangalore, 560029 Karnataka India

**Keywords:** DMD, MLPA, PROVEAN, Hydrophobicity profile, *eDystrophin*

## Abstract

**Background:**

Duchenne muscular dystrophy (DMD) and Becker muscular dystrophy (BMD) are X-linked recessive disorders caused by mutations in the DMD gene. The aim of this study was to predict the effect of gene mutations on the dystrophin protein and study its impact on clinical phenotype.

**Methods:**

In this study, 415 clinically diagnosed patients were tested for mutations by Multiplex ligation dependent probe amplification (MLPA). Muscle biopsy was performed in 34 patients with negative MLPA. Phenotype-genotype correlation was done using PROVEAN, hydrophobicity and *eDystrophin* analysis. We have utilized bioinformatics tools in order to evaluate the observed mutations both at the level of primary as well as secondary structure.

**Results:**

Mutations were identified in 75.42% cases, of which there were deletions in 91.6% and duplications in 8.30%. As per the reading frame rule, 84.6% out-of frame and 15.3% in-frame mutations were noted. Exon 50 was the most frequently deleted exon and the exon 45–52 region was the hot-spot for deletions in this cohort. There was no correlation noted between age of onset or creatine kinase (CK) values with extent of gene mutation. The PROVEAN analysis showed a deleterious effect in 94.5% cases and a neutral effect in 5.09% cases. Mutations in exon 45–54 (out of frame) and exon 46–54 (in-frame) regions in the central rod domain of dystrophin showed more negative scores compared to other domains in the present study. Hydrophobicity profile analysis showed that the hydrophobic regions I & III were equally affected. Analysis of deletions in hinge III hydrophobic region by the *eDystrophin* programme also predicted a hybrid repeat seen to be associated with a BMD like disease progression, thus making the hinge III region relatively tolerant to mutations.

**Conclusions:**

We found that, while the predictions made by the software utilized might have overall significance, the results were not convincing on a case by case basis. This reflects the inadequacy of the currently available tools and also underlines the possible inadequacy of MLPA to detect other minor mutations that might enhance or suppress the effect of the primary mutation in this large gene. Next Generation Sequencing or targeted Sanger sequencing on a case by case basis might improve phenotype- genotype correlation.

**Electronic supplementary material:**

The online version of this article (doi:10.1186/s12881-017-0431-6) contains supplementary material, which is available to authorized users.

## Background

Duchenne muscular dystrophy is the most severe and common form of X-linked recessive neuromuscular degenerative disorder affecting 1 in 3500 live male births [1]. It is clinically characterized by progressive muscle weakness, calf hypertrophy and elevated creatine kinase (CK) levels, wheel chair bound before the age of 12 and death due to respiratory failure. Becker muscular dystrophy is a milder form with an incidence of 1 in 20,000 male births [2, 3]. Both are caused due to defects in the DMD gene that encodes a 427 kDa cytoskeletal protein dystrophin located at Xp.21.2. Dystrophin is the largest human gene consisting of 79 exons which encodes a 14.6 Kb mRNA expressed mainly in skeletal muscle, heart and brain [4, 5]. Clinical severity depends on whether the reading frame is maintained. Disruption of the reading frame (out of frame) leading to prematurely truncated nonfunctional dystrophin usually gives rise to a severe DMD phenotype. Although (In-frame) mutations retaining ORF, code for semi-functional dystrophin and are predicted to be associated with a mild BMD phenotype, there are exceptions to this general rule as there are patients with severe DMD carrying in-frame mutations [5–7]. About 65% of DMD gene mutations are accounted for by intragenic deletions, 10–15% by duplications and remaining by point mutations [8]. Deletions are mostly clustered in two hotspots, either at proximal (towards 5’end) or distal (towards 3’end) part of the gene [9]. Therapeutic approaches are also designed to transform the Duchenne phenotype to milder Becker phenotype by restoring the expression of the dystrophin gene via exon - skipping strategies [10, 11]. As no effective treatment is available for DMD/BMD, an accurate genetic diagnosis for prenatal screening is very crucial. Several techniques are available to identify mutations in the dystrophin gene. Multiplex ligation dependent probe amplification (MLPA) technique can determine the chromosomal DNA copy number changes for each exon in a single multiplex - PCR based reaction. MLPA covers all 79 exons in the DMD gene and detects deletion/duplication of one or more exons in the dystrophin gene [12].

In this study, phenotype – genotype correlation was performed based on mutational findings of 415 clinically suspected DMD/BMD patients at our centre in Southern India. This paper is an attempt to understand the impact of mutations on the structure of the dystrophin protein using bioinformatics tools.

## Methods

### Subjects

Clinically suspected cases (*n* = 415) of DMD/BMD referred for genetic testing, as a part of diagnosis from August 2013 to July 2015 were included in this study. Diagnosis was based on clinical presentation, elevated CK level, pattern of inheritance and muscle biopsy. Muscle biopsy was performed in thirtyfour patients where the genetic analysis was negative. The study was approved by the Institutional Ethics committee and written informed consent was obtained from all patients.

### Genetic testing by Multiplex ligation-dependent probe amplification

Blood samples were collected in EDTA vacutainer and genomic DNA was extracted by salting out method and stored at -20 °C until tested [13]. The MLPA reaction was carried out to screen all exons of the dystrophin gene using SALSA MLPA P034 and P035 probe sets (available commercially MRC Holland, Netherlands). The procedure was performed according to manufacturer’s instructions [12]. Amplified products were separated using ABI 3500 XL Genetic analyzer and data were analyzed by coffalyser software. Normal healthy individuals were used as controls and included in every run.

### Muscle biopsy

Open muscle biopsy was performed in 34 patients under local anaesthesia after obtaining informed consent. Tissue samples were immediately frozen in isopentane precooled in liquid nitrogen. Serial 6-μm thick sections were cut using cryostat and stained for routine histological stains — hematoxylin-eosin (HE), modified Gomori trichrome and enzyme histochemical stains - NADH-tetrazolium reductase, succinic dehydrogenase, cytochrome oxidase and ATPase at PH 9.5 and 4.6.

Immunohistochemical staining using monoclonal antibodies against dystrophin (dys1, dys2, dys3) and sarcoglycans (α, β, γ, δ) as primary, and HRP – conjugated novalink polymer as secondary was carried out. All sections were compared with control samples (from patients other than muscular dystrophy) labelled in parallel.

### Bioinformatics analysis

SIFT, PolyPhen-2, Mutation Assessor, MAPP, PANTHER, Condel and several others are the computational methods developed based on evolutionary principles to predict the effect of coding variants on protein function. These tools focus only on single amino acid substitutions whereas, the PROVEAN (**Pro**tein **V**ariation **E**ffect **A**nalyzer) tool predicts the functional impact for all classes of protein sequence variations, not only single amino acid substitutions, but also insertions, deletions, and multiple substitutions (http://provean.jcvi.org). The PROVEAN tool was applied to generate a PROVEAN score for each variant. This score can be used as a measure to distinguish disease variants and common polymorphisms. This tool was used in this study to predict the functional effects of protein sequence variations (deletion/duplication) [14].

Hydrophobicity profile analysis was also carried out. Dystrophin protein sequence was obtained from Genbank (http://www.ncbi.nlm.nih.gov/genbank) and imported into Bioedit software 7.0.1. Kyte-Doolittle scale mean hydrophobicity profile analysis was performed to construct the hydrophobic regions of dystrophin protein to find out whether mutation in the hydrophobic regions has a role in pathogenesis of DMD [15].

*eDystrophin* database (http://edystrophin.genouest.org) was used to analyze the consequences of in-frame mutations in BMD patients on dystrophin protein in this cohort. It provides three-dimensional structure model of the mutation site and changes in the interacting partners of the protein due to mutation [16].

## Result

### Clinical findings

Totally 415 clinically suspected cases of DMD/BMD were subjected to MLPA testing. Most of the patients had delayed milestones, difficulty in climbing stairs and rising from the floor. The mean age of onset for DMD & BMD were 4.40 ± 2.30 years and 12.53 ± 6.55 years respectively. The mean age at presentation was 9.72 ± 6.36 years and the mean creatine kinase value was 11218.9 ± 9799 U/L. Family history of DMD/BMD was observed in 18.5% of cases. Contractures were common and observed in 64.6% of cases. There were thirty patients in this cohort who were wheel chair dependent at an average age of 9.5 years. Intelligence quotient performed in 30 patients using Binet Kamat scale showed average intelligence in 15 (50%), dull normal in 6(20%), mild mental retardation in 3 (10%) and borderline intelligence in 3 (10%) respectively.

### Genetic findings

**Fig. 1:**
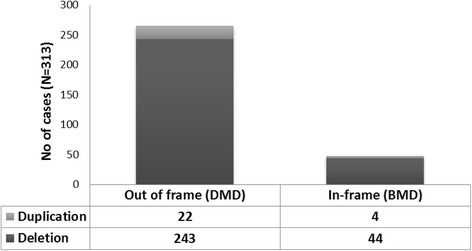
Identified mutations & their phenotype

**Fig. 2:**
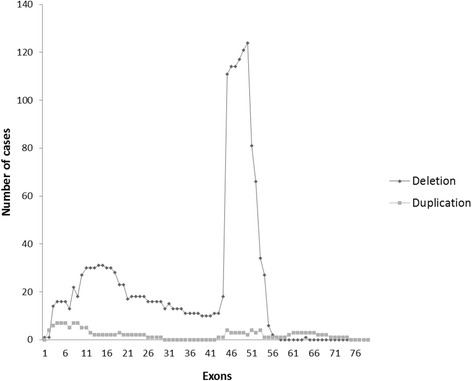
Mutational frequency of each exon in the DMD gene

Out of 415 cases, mutations were found in 313 (75.42%) by MLPA testing. Among 313 cases, 265 (84.6%) showed out of frame mutations (DMD) and 48 (15.33%) cases showed in-frame mutations (BMD) [Fig. 1]. Clinically, 284 had been suspected to have DMD and 29 to have BMD. Deletions were observed in 287 cases (91.6%) and duplications in 26 cases (8.30%). Distal deletions accounted for 74.2%, proximal were 16.56%, while nine cases showed both proximal and distal mutations. Single exon deletions were identified in 79/313 cases (25.23%) of which exon 45 and 51 were commonly deleted. The hot spot regions were exons 45–52 (17/263), exons 45–50 (16/263) and exon 46–47 (10/263). Multi-exon deletions (>25 exons) were observed in 9 cases, of which 3 cases showed severe DMD phenotype [Table 1]. Overall, exon 50 was the most frequently deleted followed by exons 49, 48, 47 & 46. Large duplications (>15 exons) were observed in 3 out of 26 cases. The most frequently duplicated single exon was from the exon 4–9 region. Overall, distal region exons were more frequently duplicated than proximal. Figure 2 shows the rearrangement frequency of each exon in the DMD gene.

**Table 1:** MLPA results of Multiexon deletion (> 25) and missing amino acids with the predicted PROVEAN scores. IF/OF (In frame/ out of frame)

S.No	Case no	Diagnosis	MLPA results	OF/IF	Protein deleted	Pathogenicity score
1	P1	DMD	Exon 8–47 deletion	OF	D217_L2255del	−3506.484
2	P3	DMD	Exon 17–52 deletion	OF	I665_R2553del	−3407.885
3	P4	DMD	Exon 8–41 deletion	OF	D217_I1974del	−3205.69
4	P9	DMD	Exon 11–41 deletion	IF	G384_I1974del	−3138.91
5	P10	BMD	Exon 14–42 deletion	IF	V535_K2039del	−2953.782
6	P11	DMD	Exon 3–34 deletion	IF	F32_V1559del	−2804.117
7	P12	DMD	Exon 3–34 deletion	IF	F32_V1559del	−2804.117
8	P13	DMD	Exon 11–31 deletion	IF	G384_Q1448del	−2374.516
9	P16	DMD	Exon 3–25 duplication	IF	F32_Q1144del	−2020.217

The maximum number of In-Frame mutations showed exon 45–47 deletion (29.4%) followed by exon 45–48 deletion (12.5%). Majority of in-frame mutations were in the distal region (68.6%) as compared to the proximal (31.3%) region.

### Immunohistochemical findings

**Fig. 3:**
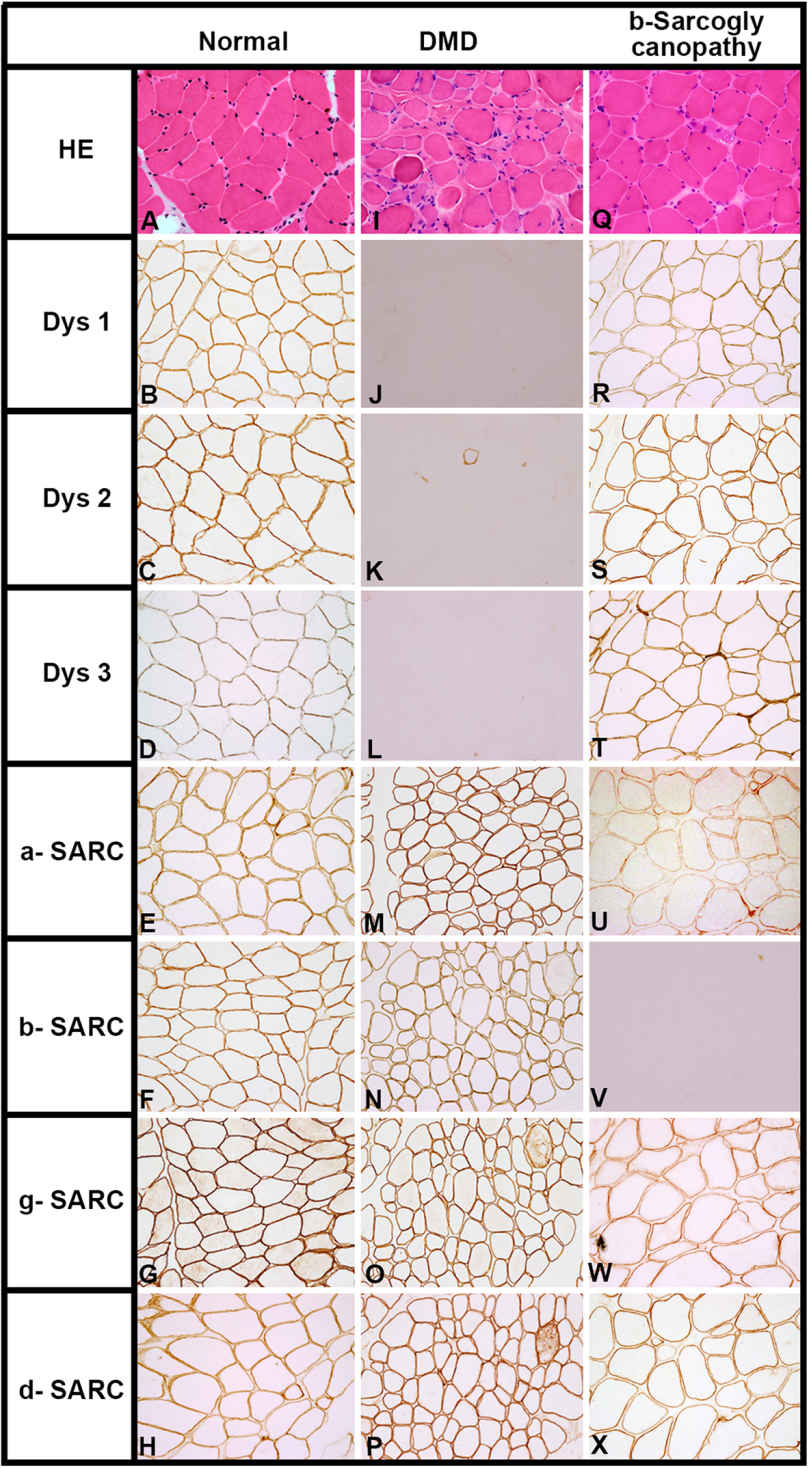
Transversely cut skeletal muscle tissue shows dystrophic features on HE staining in both DMD and β- sarcoglycanopathy (Fig I & Q) as against normal muscle tissue (Fig A). Immunohistochemically, antibodies against dystrophin (dys1,2,3) and sarcoglycans (α,β,γ,δ) shows preserved expression along the membrane in all the fibres (Fig B-H) in normal muscle tissue, while total loss of expression for dystrophin (Fig J,K,L) and preserved expression for sarcoglycans (Fig M,N,O,P) indicates the diagnosis of the DMD. Note: Preserved expression of dystrophin (Fig R,S,T) and δ &γ sarcoglycans (Fig W,X) reduced α-sarcoglycan (Fig U)and complete absence of β-sarcoglycan (Fig V) in a case of b-sarcoglycanopathy

**Fig. 4:**
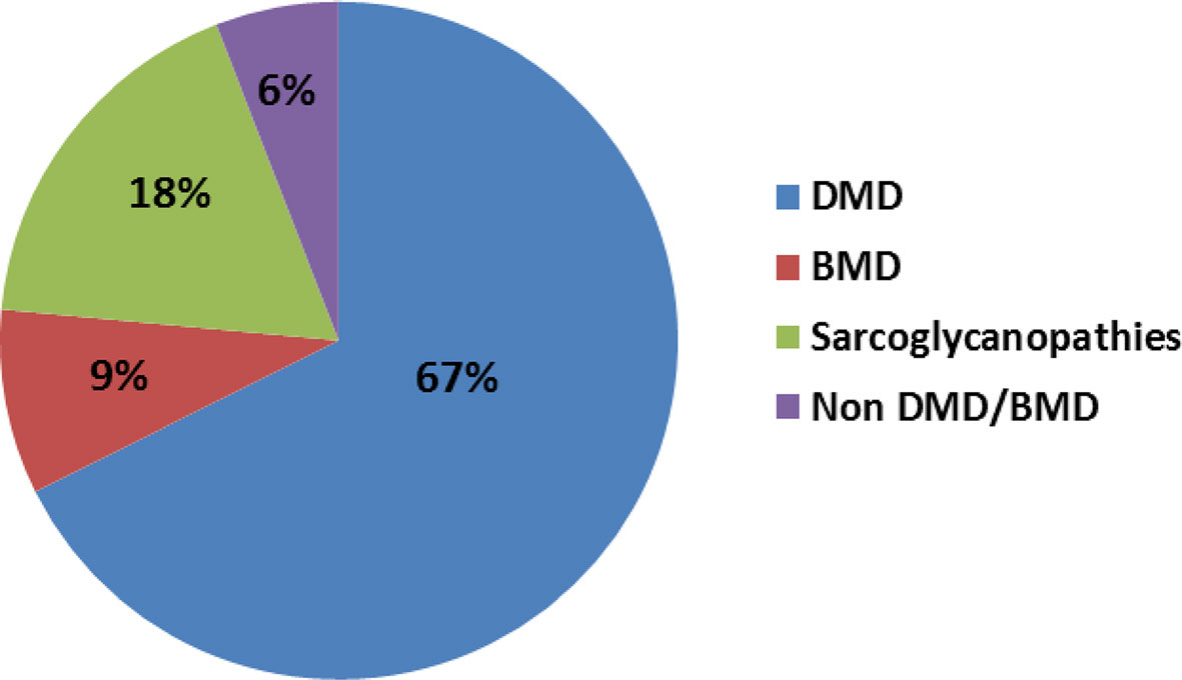
MLPA negative cases analysed by immunostaining (*n* = 34)

Muscle biopsy performed in 34 of the 102 MLPA negative cases showed dystrophic features on routine histological stains. Immunostaining showed complete loss of dystrophin expression in 23/34 (67%) cases, reduced and patchy dystrophin expression at least on one domain in 3/34 (9%) cases, sarcoglycan (α,β,γ,δ) deficiency in 4/34 cases (12%), β sarcoglycan deficiency in 1/34 cases (3%), α,β sarcoglycan deficiency in 1/34 cases (3%) and no deficiency in 2/34 cases (6%) [Figs. 3 and 4].

### PROVEAN analysis

**Fig. 5:**
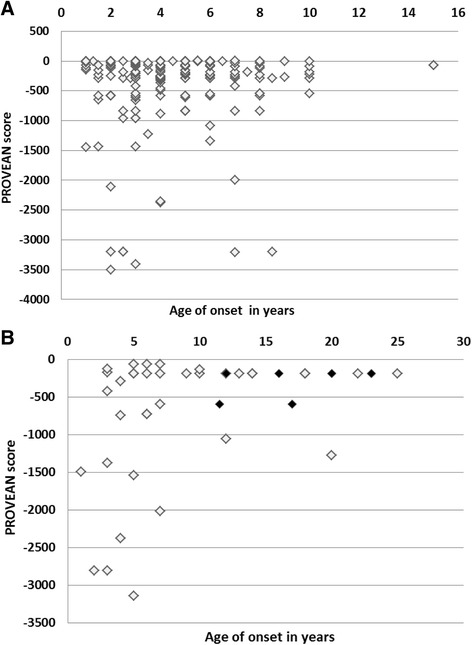
Distribution of PROVEAN score on dystrophin protein (**a**) Effect of Out of frame mutation on dystrophin protein, (**b**) Effect of In-frame mutation on dystrophin protein. *Variants with a score equal to or below -2.5 are considered ‘deleterious’ *Variants with a score above -2.5 are considered ‘neutral’ ♦ Region with hinge III deletion (**b**)

The possible biological functional effect of sequence variations on the dystrophin protein was tested for 313 cases by PROVEAN analysis. The output consisted of a PROVEAN score and a prediction of ‘deleterious’ or ‘neutral’ based on the magnitude of the score and a set threshold of (-2.5) . Deleterious effect was observed in 297 (94.5%) cases and neutral effect in 16 (5.09%) cases. Further examination of the neutral effect mutations which included both out of frame and in-frame mutations revealed the deletions to be either exon 51 deletion/duplication or duplications in exon 2–7, 2–11 region in our cases. Figure 5 shows a graph of PROVEAN score plotted against age of onset.

### Hydrophobicity profile analysis

Kyte-Doolittle scale mean hydrophobicity profile analysis was performed for 48 cases with in-frame mutation. Mutational disruption in the hydrophobic regions I & III was found in 7 cases each. In this group of cases, hydrophobic region I & III was equally affected. Table 2 represents the Dystrophin hydrophobic regions mutations.

**Table 2:** Hydrophobic region mutations identified in this cohort by Kyte-Doolittle scale mean hydrophobicity profile analysis using BioEdit software

Hydrophobic region	No. of cases with in-frame mutation (*n* = 48)
Not involved	33
Involved	15
*Region I*	7
*Region II*	1
*Region III*	7
*Region IV*	0

### *eDystrophin* analysis

**Fig. 6:**
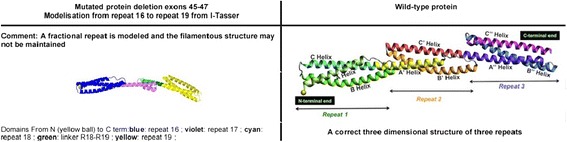
3D- structure model of the mutation site of exon 45–47 deletion obtained from (http://edystrophin.genouest.org)

Using *eDystrophin* database, we analyzed consequences of in-frame mutations on dystrophin protein structure for 44 available mutations. On 3D structure modelling of the dystrophin protein, 12 cases retain the typical filamentous structure of dystrophin, while the filamentous structure was not maintained in 25 cases. We found that mutations between exon 1–30 did not affect the protein structural domains (7 out of 44 cases). Figure 6 depicts the effect of the most frequent in-frame mutation exon 45–47 deletion in our sample.

## Discussion

This study presents the retrospective analysis of genetic testing for 415 clinically suspected DMD/BMD patients in our centre located in southern India using MLPA. MLPA is a rapid and highly sensitive technique used to detect deletions and duplications in the DMD gene [17–21]. In this cohort, the overall detection rate by MLPA was 75.42%. Our findings are comparable to the study Wang et al., [22], who reported a mutation rate of 72.5% in the Chinese population. The present study showed deletions in 91.6% cases and duplications in 8.30% cases in the dystrophin gene. The frequency of deletion was more common than duplications, similar to frequency reported from other parts of India [23–27]. The reported deletion rates in Pakistanis is 40.7%, Chinese 66.25%, Korean 45.5% and in Taiwanese patients 36%, thus showing possible variations among different populations [22, 28–30]. The duplication rate in our cases mainly involved larger fragments and the pattern of duplication was more towards the distal part of the gene unlike other populations [22]. Random age distribution was observed in this cohort, i.e. there was no correlation between extent of deletion/duplication or position of mutation, and the age of onset of clinical symptoms. This finding was similar to the Dubowitz study where no correlations could be drawn between age of onset or severity to the extent of mutation [31].

Muscle biopsy was undertaken for patients who tested negative by MLPA. Immunohistochemically, the diagnosis of DMD was established for the patients with complete absence of staining along the sarcolemma. However, BMD patients showed heterogenous dystrophin expression ranging from reduced patchy staining to normal staining on IHC [32–34]. The dystrophin – glycoprotein complex is responsible for stabilizing the muscle fiber, a perturbation in any of its components may result in overlapping clinical presentation. Six patients with suspected DMD showed normal dystrophin labelling, but absence of sarcoglycans expression. Immunohistochemistry thus still remains the gold standard method for diagnosing muscular dystrophies [24]. IHC should be considered to detect dysfunctional dystrophin expression when genetic testing results are negative.

### Genotype- Phenotype correlation

Age of onset, CK values, age at wheel chair bound and IQ score was evaluated in this study to define genotype and clinical phenotype correlation. Patients who lost ambulation at an average age of 9.5 years were seen to have deletions in the exon 45–55 region of the DMD gene (*n* = 30). A lower IQ score was noted largely in patients who had distal gene deletions. This was keeping with expectation as the full length isoform Dp427 is minimally expressed in the brain [35]. The dystrophin isoforms Dp140 & Dp71 which are highly expressed in the brain lack the proximal exons. The role of dystrophin in the brain remains unclear, however mutations at the 3’ end of the gene have been associated with compromised brain function. Ricotti et al [36] observed that mutations disrupting the isoform Dp140 & Dp70 are more frequently associated with lower IQ scores. There was no correlation noted in CK values with gene mutation as this was a cross sectional study [37].

The PROVEAN analysis predicts effect of mutation based on the changed aminoacid sequence of mutated dystrophin protein. Mutations in exon 45–54 (Out of frame) and exon 46–54 (In-frame) region in the central rod domain of dystrophin showed more negative scores compared to other domains in the present study. Previous reports demonstrated that the phosphorylation sites of dystrophin present within the central rod domain including T2621 which is encoded by exon 53 might affect the structure of this N terminal domain. Dystrophin upon phosphorylation is believed to undergo a conformational change in the N-terminal actin binding domain, thereby enhancing its affinity for myofibrillar actin [38, 39]. Actin also binds the central rod domain encoded by exon 31–45 which is located between spectrin type repeats 11–17 [40]. This reconfirms the role of rod domain in dystrophin function [41].

Dystrophin protein interacts with integral membrane proteins to form the dystrophin- glycoprotein complex (DGC). The role of DGC is to stabilize the sarcolemma and protect the muscle fibers from long term damage. The hydrophobic region of dystrophin plays an important role in maintaining the stability and interaction with other proteins. There are four hydrophobic regions in dystrophin coded by exons 3–6 (region I), 42 (region II), 51 (region III), and 65–68 (region IV) which are found on the calponin homology CH2 domain on the actin-binding domain (ABD), spectrin-type repeat 16, hinge III and the EF Hand domain respectively. Liang et al [16] observed that mutational disruption in the hydrophobic region I, II, IV directly impairs the DGC function which leads to severe DMD phenotype, whereas, region III disruption leads to a less severe BMD phenotype. Carsana et al [42] demonstrated that an in-frame deletion of the hinge region in the distal rod domain shows a milder phenotype compared with deletions that do not include hinge III region. Further analysis by PROVEAN programme showed the deletion of hinge III region has more negative score compared to deletions which do not include the hinge III region. This suggests that clinical severity of the BMD maybe determined by the presence or absence of hinge III region in the dystrophin protein. However, all patients (*n* = 12) with exon 51 deletion /duplication corresponding to region III with age of onset ranging from 1–8 years had a severe DMD phenotype as predicted by reading frame rule.

Dystrophin is a large cytoskeletal protein comprised of four domains. The larger central rod domain has 24 repeating units similar to spectrin-like repeats. The repeat is a triple coiled coil structure made up of three helices with heptad pattern of amino acids [43, 44]. This filamentous protein acts as a scaffold for several interacting partners and also provides resistance to the stress of muscle contraction. Any mutation altering this structure of dystrophin might be expected to affect its function along with that of its binding partners. The *eDystrophin* programme provides a computational model for each in-frame mutation and shows whether an approximate 3D filamentous structure is reconstituted (hybrid repeat) or a more deleterious structure (fractional) repeat is formed. Nicholas et.al [44] reported the differences in the structure of mutant dystrophin protein may be responsible for clinical heterogeneity in BMD patients. They observed earlier wheel chair dependency and early development of cardiomyopathy in patients with exon 45–47 (Fractional repeat) deletion compared to exon 45–48 (Hybrid repeat) deletion. Fractional repeat has slower refolding dynamics and higher molecular surface hydrophobicity compared to hybrid repeat. In this study, the most prevalent in-frame deletion observed was exon 45–47 deletion which was associated with age of onset 4–20 years and exon 45–48 deletion which was associated with age of onset 5–20 years. Analysis of hinge III deletion in *e-dystrophin* programme also results in retention of typical filamentous structure of dystrophin (hybrid repeat). The hybrid repeat reconstitution depends on exon phasing and though the presence of hybrid repeat does not restore the dystrophin function completely, it results in a more functional protein compared to fractional repeat [15]. Exon phasing if considered along with restoration of reading frame for exon-skipping therapy might result in improved clinical outcome.

To assess the effect of mutation on clinical severity, we did correlations between pathogenicity score and the age of onset of the clinical symptoms primarily, observed muscle weakness. Both DMD & BMD patients showed no definite correlation between sequence variation as assessed by PROVEAN score and clinical symptoms. In this cohort, we observed ‘neutral effect’ both in patients having exon 51 deletion/duplication which would produce truncated protein and duplications in exon 2–11 region, where the entire amino acid sequence is disturbed. We hypothesize that this mild phenotype seen as milder disease progression despite a large predicted ‘out of frame’ mutation in the proximal part of the protein could be due to compensatory changes in the downstream region. Further, the possibility of false positive deletion calls due to variations at the site of primer binding cannot be ruled out. These mutations which cannot be detected by MLPA should be further evaluated by sequencing.

## Conclusion

In this study, the mutational spectrum of patients at this centre were compared with global populations. Our data reiterates that muscle biopsy followed by immunohistochemistry should be considered only when genetic tests results are negative. The phenotype– genotype correlation revealed that the clinical severity of BMD depends on the site and type of deletion to some extent. It also indicates that the presence of central rod domain plays an important role in dystrophin function and disease progression of DMD/BMD. Identification and characterization of dystrophin domains and their binding partners is very important for understanding the pathways that are involved, which in turn might help in devising treatments for this devastating disorder. An accurate genetic diagnosis is essential for genetic counselling and patient’s treatment because therapies are mutation-specific. It may be advisable to carry out targeted sequencing to detect point mutations or any additional variants that may affect disease severity.

## Additional file

Additional file 1: Listing of mutations found in our samples. (XLSX 16 kb)

## Abbreviations

ABD: Actin binding domain
BMD: Becker’s muscular dystrophy
CK: Creatine kinase
DGC: Dystrophin glycoprotein complex
DMD: Duchenne muscular dystrophy
HE: Hemotoxylin eosin
HRP: Horseradish peroxidase
IHC: Immunohistochemistry
MLPA: Muliplex ligation dependent probe amplification
PROVEAN: Protein variation effect analyzer

## References

[1] Emery AE (1998). The Muscular Dystrophies. BMJ.

[2] Emery AE (1991). Population frequencies of inherited neuromuscular diseases—a world survey. Neuromuscular Disord.

[3] Bushby KM, Thambyayah M, Gardner-Medwin D (1991). Prevalence and incidence of Becker muscular dystrophy. Lancet.

[4] Hoffman EP, Brown RH, Kunkel LM (1987). Dystrophin: the protein product of the Duchenne muscular dystrophy locus. Cell.

[5] Monaco AP, Bertelson CJ, Liechti-Gallati S, Moser H, Kunkel LM (1988). An explanation for the phenotypic differences between patients bearing partial deletions of the DMD locus. Genomics.

[6] Muntoni F, Torelli S, Ferlini A (2003). Dystrophin and mutations: One gene, several proteins, multiple phenotypes. Lancet Neurol.

[7] Aartsma-Rus A, Van Deutekom JC, Fokkema IF, Van Ommen GJ, Den Dunnen JT (2006). Entries in the Leiden Duchenne muscular dystrophy mutation database: an overview of mutation types and paradoxical cases that confirm the reading-frame rule. Muscle Nerve.

[8] Koenig M, Beggs AH, Moyer M, Scherpf S, Heindrich K, Bettecken T (1989). The molecular basis for Duchenne versus Becker muscular dystrophy: Correlation of severity with type of deletion. Am J Hum Genet.

[9] Blake DJ, Weir A, Newey SE, Davies KE (2002). Function and genetics of dystrophin and dystrophin-related proteins in muscle. Physiol Rev.

[10] Cirak S (2011). Exon skipping and dystrophin restoration in patients with Duchenne muscular dystrophy after systemic phosphorodiamidate morpholino oligomer treatment: An open-label, phase 2, dose-escalation study. Lancet.

[11] Wood MJ, Gait MJ, Yin H (2010). RNA-targeted splice-correction therapy for neuromuscular disease. Brain.

[12] Schouten JP, McElgunn CJ, Waaijer R, Zwijnenburg D, Diepvens F, Pals G (2002). Relative quantification of 40 nucleic acid sequences by multiplex ligation-dependent probe amplification. Nucleic Acids Res.

[13] Miller SA, Dykes DD, Polesky HF (1988). A simple salting out procedure for extracting DNA from human nucleated cells. Nucleic Acids Res.

[14] Choi Y, Sims GE, Murphy S, Miller JR, Chan AP (2012). Predicting the Functional Effect of Amino Acid Substitutions and Indels. PLoS One.

[15] Nicolas A, Lucchetti-Miganeh C, Ben YR, Kaplan J-C, Chelly J, Leturcq F (2012). Assessment of the structural and functional impact of in-frame mutations of the DMD gene, using the tools included in the eDystrophin online database. Orphanet J Rare Dis.

[16] Liang Y, Chen S, Zhu J, Zhou X, Yang C, Yao L, Zhang C (2015). Dystrophin hydrophobic regions in the pathogenesis of Duchenne and Becker muscular dystrophies. Bosn J Basic Med Sci.

[17] Stuppia L, Antonucci I, Palka G, Gatta V (2012). Use of the MLPA assay in the molecular diagnosis of gene copy number alterations in human genetic diseases. Int J Mol Sci.

[18] Janssen B, Hartmann C, Scholz V, Jauch A, Zschocke J (2005). MLPA analysis for the detection of deletion, duplication and complex arrangements in the dystrophin gene: potential and pitfalls. Neurogenetics.

[19] Lai KS, Lo IF, Tong TM, Cheng LY, Lam ST (2006). Detecting exon deletions and duplications of the DMD gene using Multiplex Ligation-dependent Probe Amplification (MLPA). Clin Biochem.

[20] Prior TW, Bridgeman SJ (2005). Experience and strategy for the molecular testing of Duchenne muscular dystrophy. J Mol Diag.

[21] Gatta V, Scarciolla O, Gaspari AR, Palka C, De Angelis MV, Di Muzio A, Guanciali-Franchi P, Calabrese G, Uncini A, Stuppia L (2005). Identification of deletions and duplications of the DMD gene in affected males and carrier females by multiple ligation probe amplification (MLPA). Hum Genet.

[22] Wang X, Wang Z, Yan M, Huang S, Chen TJ, Zhong N (2008). Similarity of DMD gene deletion and duplication in the Chinese patients compared to global populations. Behav Brain Funct.

[23] Swaminathan B, Shubha GN, Shubha D, Murthy AR, Kiran Kumar HB, Shylashree S, Gayathri N, Jamuna R, Jain S, Purushottam M, Nalini A (2009). Duchenne muscular dystrophy: a clinical, histopathological and genetic study at a neurology tertiary care center in Southern India. Neurol India.

[24] Manjunath M, Kiran P, Preethish-Kumar V, Nalini A, Singh RJ, Gayathri N (2015). A comparative study of mPCR, MLPA, and muscle biopsy results in a cohort of children with Duchenne muscular dystrophy: A first study. Neurol India.

[25] Mallikarjuna Rao GN, Hussain T, Geetha Devi N, Jain S, Chandak GR, Ananda Raj MP (2003). Dystrophin gene deletions in South Indian Duchenne muscular dystrophy patients. Indian J Med Sci.

[26] Khalap NV, Joshi VP, Ladiwalla U, Khadilkar SV, Mahajan SK (1997). A report on higher frequency of DMD gene deletion in the Indian subcontinent. Indian J Hum Genet.

[27] Nadkarni JJ, Dastur RS, Viswanathan V, Gaitonde PS, Khadilkar SV (2008). Duchenne and Becker muscular dystrophies: An Indian update on genetics and rehabilitation. Neurol India.

[28] Hassan MJ, Mahmood S, Ali G, Bibi N, Waheed I, Rafiq MA (2008). Intragenic deletions in the dystrophin gene in 211 Pakistani Duchenne muscular dystrophy patients. Pediatr Int.

[29] Lee BL, Nam SH, Lee JH, Ki CS, Lee M, Lee J (2012). Genetic analysis of dystrophin gene for affected male and female carriers with Duchenne/ Becker muscular dystrophy in Korea. J Korean Med Sci.

[30] Hwa HL, Chang YY, Chen CH, Kao YS, Jong YJ, Chao MC (2007). Multiplex ligation-dependent probe amplification identification of deletions and duplications of the Duchenne muscular dystrophy gene in Taiwanese subjects. J Formo Med Assoc.

[31] Dubowitz V (1995). Muscle disorders in childhood.

[32] Na SJ, Kim WJ, Kim SM, Lee KO, Yoon B, Choi YC (2013). Clinical, immunohistochemical, Western blot, and genetic analysis in dystrophinopathy. J Clin Neurosci.

[33] Beggs AH, Hoffman EP, Snyder JR, Arahata K, Specht L, Shapiro F (1991). Exploring the molecular basis for variability among patients with Becker muscular dystrophy: Dystrophin gene and protein studies. Am J Hum Genet.

[34] Werneck LC, Scola RH, Maegawa GH, Werneck MC (2001). Comparative analysis of PCR-deletion detection and immunohistochemistry in Brazilian Duchenne and Becker muscular dystrophy patients. Am J Med Genet.

[35] Magri F, Govoni A (2011). Genotype and phenotype characterization in a large dystrophinopathic cohort with extended follow-up. J Neurol.

[36] Ricotti V, Mandy WP, Scoto M (2016). Neurodevelopmental, emotional, and behavioural problems in Duchenne muscular dystrophy in relation to underlying dystrophin gene mutations. Dev Med Child Neurol.

[37] Bastaki LA (1999). Genotype-Phenotype Correlation among patients with Dystrophinopathies. Alexandria J Pediatrics.

[38] Luise M, Presotto C, Senter L, Betto R, Ceoldo S, Furlan S, Salvatori S, Sabbadini RA, Salviati G (1993). Dystrophin is phosphorylated by endogenous protein kinases. Biochem J.

[39] Senter L, Ceoldo S, Petrusa MM, Salviati G (1995). Phosphorylation of dystrophin:effects on actin binding. Biochem Biophys Res Commun.

[40] Yokota T, Duddy W, Partridge T (2007). Optimizing Exon Skipping Therapies for DMD. Acta Myologica.

[41] Harper SQ, Hauser MA, DelloRusso C, Duan D, Crawford RW, Phelps SF, Harper HA, Robinson AS, Engelhardt JF, Brooks SV, Chamberlain JS (2002). Modular flexibility of dystrophin: implications for gene therapy of Duchenne muscular dystrophy. Nat Med.

[42] Carsana A, Frisso G, Tremolaterra MR, Lanzillo R, Vitale DF, Santoro L (2005). Analysis of dystrophin gene deletions indicates that the hinge III region of the protein correlates with disease severity. Ann Hum Genet.

[43] Menhart N: Hybrid spectrin type repeats produced by exon-skipping in dystrophin. Biochem Biophys Acta 2006, 1764:993-999. 10.1016/j.bbapap.2006.03.017 PMC1925050 16716778

[44] Nicolas A, Raguénès-Nicol C, Ben Yaou R, Ameziane-Le Hir S, Chéron A, Vié V, Claustres M, Leturcq F, Delalande O, Hubert JF, Tuffery-Giraud S, Giudice E, Le Rumeur E, French Network of Clinical Reference Centres for Neuromuscular Diseases (CORNEMUS) (2015). Becker muscular dystrophy severity is linked to the structure of dystrophin. Hum Mol Genet.

